# Molecular Insights into Adsorption Mechanisms of Micro- and Nanoplastics on Effective Adsorbent Materials

**DOI:** 10.3390/molecules31173089

**Published:** 2026-09-03

**Authors:** Angelo Fenti, Pasquale Iovino

**Affiliations:** 1 Department of Advanced Medical and Surgical Sciences, University of Campania “Luigi Vanvitelli”, 80138 Naples, Italy; 2 Department of Environmental, Biological and Pharmaceutical Science and Technologies, University of Campania “Luigi Vanvitelli”, 81100 Caserta, Italy; 3 Center for Environmental Pollution and Cardiovascular Diseases, University of Campania “Luigi Vanvitelli”, 80138 Naples, Italy

**Keywords:** microplastics, nanoplastics, adsorption mechanisms, adsorbent materials, water remediation, wastewater treatment

## Abstract

Existing reviews on micro- and nanoplastic (MNP) removal from water rarely link adsorbent structural features to the molecular interactions governing removal performance. This review addresses this gap by examining MNP adsorption from a mechanism-oriented perspective, mapping six canonical interaction pathways across five adsorbent classes. Adsorption emerges as a system-dependent process governed by the interplay between polymer properties and surface chemistry rather than by the material alone. Interactions such as π–π stacking and hydrophobic affinity dominate for non-functionalized polymers on carbon-rich surfaces, while electrostatic forces and hydrogen bonding become more relevant for oxidised particles. Pore structure becomes significant when particle size and porosity match, whereas chemisorption provides a stronger and faster pathway in systems containing reactive metal sites. Across material classes, differences relate more closely to scalability and sustainability than to intrinsic adsorption capacity. Bio-based materials offer a favourable balance between performance and practical implementation, while more advanced systems provide greater control but remain limited by synthesis complexity. Laboratory capacities often overestimate real performance, and removal efficiency in complex matrices is a more reliable metric. Future progress will depend on improved standardisation, integration with modelling, and validation under realistic conditions to support the transition from laboratory studies to practical applications.

## 1. Introduction

Plastic pollution is now recognized as a major global environmental challenge due to its pervasive distribution across ecosystems. According to previous research, global plastic production currently exceeds 400 million tonnes per year, while an estimated 4.8–12.7 million tonnes of plastic waste enter the oceans annually from land-based sources [1]. If present trends persist, nearly 12,000 million tonnes of plastic waste can eventually accumulate in landfills or in the natural environments by 2050 [2]. After being introduced into the environment, plastic waste gradually breaks down into smaller parts due to mechanical, physical, and biological degradation, resulting in the generation of microplastics (MPs) and nanoplastics (NPs) [3,4,5]. These particles have been detected in a wide range of environmental compartments, including marine and freshwater systems, soils, polar regions, and the atmosphere, as well as increasingly in food and drinking water systems [6,7,8]. This widespread and cross-compartment distribution ultimately leads to human exposure and accumulation, as evidenced by a growing number of studies reporting the presence of plastic particles in human tissues such as blood, lungs, placenta, and the cardiovascular system, among others [9,10,11]. Their persistence, small size, and role as carriers of contaminants make them complex pollutants that combine conventional micropollutant behaviour with emerging and still poorly understood toxic effects [12,13,14].

Conventional wastewater treatment plants (WWTPs) can remove a substantial proportion of MPs through existing treatment processes, but their effectiveness generally decreases for particles below 10 µm and for NPs. In turn, the remaining quantity may remain unfiltered and enter the water supply system as a part of the effluent or settle as a result of biological accumulation in sewage sludge [3,15]. Among others, membrane filtration and advanced oxidation processes (AOPs) have been explored as polishing steps following tertiary treatment; however, each presents intrinsic limitations. Membrane technologies, although effective, are constrained by membrane fouling and high operational and energy costs [16,17]; conversely, AOPs have been known to be energy-intensive and can lead to the creation of secondary products [18,19].

In this context, adsorption has emerged as a promising approach, combining comparatively low operating costs, typically an order of magnitude below those of activated carbon for biomass-derived sorbents, with versatility for application within current systems and flexibility in enabling rational material design aimed at particular populations of MPs and NPs [4,20]. This potential is already supported by field-level evidence, with engineered sorbents operated for one year in a semi-operational unit at a real WWTP achieving removal efficiencies above 90% [21], while laboratory-scale process trains combining adsorption and pre-filtration have also demonstrated efficient MP removal [22].

There has been an exponential rise in the literature regarding adsorbents that can selectively remove MNPs from water systems in recent years. A bibliometric analysis of 771 documents (2017–2024) [3] illustrates how rapidly research activity is growing and how diverse the materials being explored have become, including carbon-based, biopolymer-based, and metal-based adsorbents. Examples include activated carbon, graphene oxide materials, biopolymeric sponges, layered double hydroxides, and metal–organic frameworks [3,23,24]. In parallel, the study of the interaction mechanisms between MNPs and adsorbents has also seen great advancements, transitioning from being primarily qualitative in nature to being more mechanistically understood, thanks largely to the growing availability of experimental data and theories, including density functional theory (DFT) [25,26]. As a result, the field has reached a level of maturity where a purely material-based or catalogue-style overview is no longer sufficient to capture the key drivers of adsorption performance.

Existing reviews on MNP adsorption are usually organised around adsorbent classes, treatment processes, or bibliometric clusters, rather than around interaction mechanisms applied consistently across material types. Several recent studies have mapped removal technologies and adsorbent development through large-scale keyword and bibliometric analyses [3], while others have classified engineered adsorbents according to material type, generally discussing adsorption mechanisms within individual categories rather than systematically comparing them across classes [4]. Additional reviews have focused on specific material families, such as bioadsorbents [8] and porous materials [20], on individual treatment stages [17], or more broadly on PFAS removal technologies [14]. Another recent review treats adsorption mechanisms and adsorbent classes as separate surveys, without linking specific mechanisms to specific material classes, focusing instead on external operating factors and on the underexplored potential of column scale, continuous flow adsorption [27]. A mechanism-first structure has also been proposed, though only within a single material class [28]. Table 1 compares the organising principle and mechanistic scope of these reviews. Building on this gap, the present review adopts a mechanism-first perspective, applying six canonical interaction pathways, namely hydrophobic interaction, van der Waals forces, π–π stacking, electrostatic interactions, hydrogen bonding, and chemisorption, consistently across five adsorbent classes (carbon-based, bio-based, engineered porous, MOF/COF-based, and metal-based materials). Where possible, the type of evidence supporting each proposed mechanism, whether computational, spectroscopic, or kinetic, is also indicated. The scope of this review is limited to molecular-scale adsorption mechanisms in aqueous batch systems; MNP toxicology, human health effects, column or continuous flow engineering, and non-adsorptive removal technologies are mentioned only for context. The remainder of the review is organised as follows: Section 2 introduces MNP properties relevant to adsorption; Section 3 examines the six interaction mechanisms; Section 4 discusses the five adsorbent classes; Section 5 addresses comparative performance, cost, scalability and sustainability; Section 6 concludes with future directions.

## 2. MNPs in Water: Properties Relevant to Adsorption

MPs are commonly defined as plastic particles with diameters between 5 mm and 1 µm, whereas NPs are usually described as particles with diameters smaller than 1000 nm [29,30]. The polymer composition of environmental MNP populations largely mirrors global production trends, with polyethylene (PE), polypropylene (PP), polystyrene (PS), poly(ethylene terephthalate) (PET), and polyvinyl chloride (PVC) dominating and often accounting for the majority (typically 70–90%) of the total plastic mass observed in surface waters, sediments, and wastewater effluents. Other polymers, additives, and engineered fibers contribute to the remaining fraction [8,31]. Morphology of particles includes spheres, fragments, films, and fibers, while the particle size distribution favors smaller sizes, where particles less than 1 mm make up the majority of the population, although their share of the total mass is low.

Three primary parameters govern the adsorption behaviour of plastics in remediation contexts. First, MNPs are intrinsically hydrophobic (water contact angle ~90–110°), promoting interactions driven by hydrophobic and van der Waals forces [4,31]. Second, surface charge plays a critical role, since pristine MNPs carry a slightly negative ζ-potential due to ion sorption, which becomes more negative in aged particles as oxidation and biofouling introduce oxygen-containing functional groups (carboxyl, hydroxyl, and carbonyl) [8]. Third, secondary MNPs bearing additional functional groups, such as amino- or carboxyl-functionalized PS, poly(methyl methacrylate) (PMMA), or PVC, show enhanced affinity for charged and polar adsorbents [3,4].

A further aspect concerns the role of MNPs as carriers of co-pollutants. Due to their high surface-to-volume ratio, MPs and NPs can adsorb significant amounts of hydrophobic organic contaminants, pharmaceuticals, and heavy metals [27,32]. This behaviour appears widespread across polymer types, with a systematic review of 68 adsorption studies across 178 organic compounds showing that octanol-water partitioning can partly predict uptake, and that adsorption of persistent pollutants such as per- and polyfluoroalkyl substances (PFASs) onto polystyrene can be markedly enhanced under saline conditions [33,34]. Reported sorbates span a broad range of pollutant classes, including polychlorinated biphenyls, polybrominated diphenyl ethers, organochlorine pesticides such as DDT and hexachlorocyclohexanes, polycyclic aromatic hydrocarbons, and several pharmaceuticals and antibiotics [35]. From an adsorbent-design perspective, this dual role represents both an opportunity and a challenge, since capturing MNPs also removes the pollutants they carry, while in real water matrices the adsorbent must compete with MNPs for the same dissolved species. The effectiveness of an MNP-targeted adsorbent therefore depends critically on its selectivity in complex mixtures, a parameter that remains relatively underexplored in the literature. Selectivity effects of this kind can be substantial, as illustrated by resin studies where coexisting metal ions competed for the same binding sites, with Cr(III) reported to displace Co(II) and Ni(II) that had already been adsorbed [36]. Experimental evidence indicates that competitive effects may also play a role in MNP adsorption systems, where co-occurring contaminants, natural organic matter, and dissolved ions can influence site availability and, consequently, adsorption performance under environmentally relevant conditions [37,38].

## 3. Molecular Mechanisms

Understanding the molecular interactions between MNPs and adsorbents is essential for moving from empirical approaches to rational design, which also requires accounting for mass transfer and diffusion processes occurring within porous adsorbents [39]. Recent studies converge on six main interaction mechanisms: hydrophobic and van der Waals forces, π–π stacking, electrostatic interactions, hydrogen bonding, pore filling, and chemisorption. These mechanisms often act in combination and are influenced by environmental conditions and material properties. Their assignment is increasingly supported by molecular-scale computational evidence. Density functional theory (DFT) is a quantum-mechanical method that calculates the electronic structure of molecular systems, enabling the quantification of adsorption energies, bond formation, and charge transfer at the adsorbent surface with atomic-level precision. Molecular dynamics (MD) simulations, by contrast, describe the time evolution of atomic systems using classical force fields, allowing the decomposition of total interaction energies into their van der Waals and electrostatic components and the visualisation of adsorption trajectories at the polymer–surface interface. In the context of MNP adsorption, both approaches have proven valuable: MD captures the ensemble behaviour of polymer chains on heterogeneous surfaces, while DFT resolves the nature of specific bonds and the electronic origin of surface affinity [25,40]. In Cu-BTC/graphene oxide (GO) composites, MD simulations indicate that van der Waals forces dominate PA-66 (polyamide-66) and PU (polyurethane) adsorption, while GO enhances removal by providing additional π–π, C–H···π, and hydrogen-bonding interactions [41]. For nonpolar polyolefins such as PP on hydrophobic polydimethylsiloxane (PDMS) surfaces, 40-ns simulations demonstrate that adsorption is spontaneous and driven exclusively by van der Waals forces mediated through the methyl groups of PDMS, with the surface hydration layer offering minimal resistance, providing a finding that macroscopic batch experiments alone could not have resolved [42].

Recent experimental studies have increasingly paired DFT with laboratory data to resolve mechanistic ambiguities that spectroscopy alone cannot address. On iron-doped magnetic biochar, DFT calculations confirmed that the adsorption energy ranking CPS (carboxyl) > APS (amine) > UPS (plain) directly reflects the strength of Fe–O–polymer complexation and Fe–C bond formation, with Mayer bond orders quantifying how carboxyl and amine groups on functionalized PS enhance electron transfer to the iron-doped surface [43]. On Ni-MOF composite sponges, HOMO–LUMO energy gap analysis showed that the small ΔE_gap_ between Ni-MOF and PS facilitates electron exchange between the aromatic rings of PS and the metal framework, predicting higher removal efficiency for PS over PE and PP in precise agreement with experimental results [44]. In addition, on PET-derived activated carbon, frontier orbital calculations revealed that surface functional groups modulate adsorption energy in the order –COOH > –OH > –C=C–, and that π–π stacking between delocalized PS electrons and the sp^2^ carbon matrix dominates over electrostatic interactions when oxygenated groups are limited [45].

Taken together, these molecular-scale computational results go beyond confirming experimental trends, explaining why certain polymer–surface combinations exhibit selective adsorption, quantifying the contribution of each interaction, and offering a predictive framework that cannot be derived from macroscopic data alone. This level of mechanistic insight underpins the mechanism-by-mechanism analysis presented herein. These approaches nonetheless have intrinsic limitations. DFT calculations typically use simplified, ordered surface models at 0 K, which cannot fully capture the structural heterogeneity of real adsorbents such as biochar [46], and absolute adsorption energies vary with the exchange correlation functional and dispersion correction adopted [47]. MD simulations depend on the accuracy of the underlying force field, span timescales of nanoseconds to microseconds against experimental equilibration of minutes to hours, and cannot adequately describe chemisorptive bond formation unless reactive force fields are used [48]. Both methods therefore complement, rather than replace, experimental evidence. Fourier-transform infrared spectroscopy (FTIR) tracks shifts in characteristic vibrational bands, such as carbonyl, hydroxyl, or amine stretches, before and after adsorption [49]. X-ray photoelectron spectroscopy (XPS) resolves the binding energy and oxidation state of surface atoms, allowing new bonds and surface complexation to be identified. Zeta-potential measurements track the surface charge of MNPs and adsorbents as a function of pH and ionic strength [50]. Pseudo-first- and pseudo-second-order kinetic models are also commonly fitted to adsorption rate data to infer the underlying rate-controlling step.

Overall, the available approaches vary substantially in the strength and directness of the evidence they offer for a given mechanism. Where possible, this section indicates the level of evidence supporting each proposed mechanism. Mechanisms supported by direct approaches, such as DFT, molecular dynamics, XPS, or targeted control experiments, are noted accordingly. In contrast, mechanisms based mainly on indirect evidence, including FTIR peak shifts, zeta-potential measurements, kinetic model fitting, or functional-group considerations, should be regarded as suggestive of, rather than definitive evidence for, the proposed interaction. This is particularly relevant for pseudo-first- and pseudo-second-order kinetic fitting, often used to infer diffusion control or chemisorption, respectively. Diffusion-controlled adsorption can conform well to the pseudo-second-order equation, and data simulated under pseudo-first-order kinetics have been shown to fit the pseudo-second-order model equally well, so curve fitting alone does not reliably discriminate between the two models or unambiguously identify the underlying mechanism [51].

### 3.1. Hydrophobic and van der Waals Interactions

Hydrophobic effects constitute a major force of attraction towards MNP adsorption, especially polyolefin polymers like PE and PP as well as aliphatic portions of most commodity polymers. This effect is related to the disruption of the structured state of water at hydrophobic surfaces, which leads to an entropy-driven force of attraction that does not depend on pH [3,4]. The hydrophobic attraction force is generally supplemented by van der Waals interactions that act at molecular-level proximity between particles and adsorbents. In particular, for nonpolar polymers such as PE, which lack specific functional groups, van der Waals forces often represent a dominant interaction mechanism, as neither electrostatic nor hydrogen-bonding contributions are significant under typical environmental conditions [52,53]. Materials with intrinsically hydrophobic surfaces, such as low-oxygen activated carbon, bio-derived porous sponges, and PDMS-based coatings, exhibit high adsorption capacities toward PE and PP, as their interaction is primarily governed by rapid physisorption and hydrophobic affinity. This behaviour is further enhanced by the porous structure of the adsorbents, which promotes particle retention through non-covalent interactions and physical confinement within the pore network [3,53]. Hydrophobic interactions are generally weaker than covalent or electrostatic contributions and tend to diminish as MPs undergo environmental aging, which introduces hydrophilic oxygen-containing functional groups on their surface [8].

### 3.2. π–π Stacking

Aromatic polymers such as PS and, to a lesser extent, PET, can interact with carbon-based adsorbents via π–π stacking interactions. These interactions arise from the overlap between π-electron systems of aromatic rings and the conjugated sp^2^ domains typically found in materials such as graphene, graphene oxide, carbon nanotubes (CNTs), and high-temperature biochars [3,20,31]. These interactions represent one of the key non-covalent forces governing adsorption on carbon nanostructures and strongly influence the affinity toward aromatic compounds and polymer particles.

The interaction is relatively short-range and depends on the mutual orientation of the aromatic rings. Binding energies are typically on the order of a few kJ mol^−1^ per aromatic ring pair in model systems such as the benzene dimer, while weaker intrachain π–π interactions have been reported for aromatic polymers such as PS [54,55]. DFT studies on graphene indicate that, at larger separations, the interaction is mainly governed by dispersion forces, while closer to the equilibrium distance electrostatic contributions become more relevant [56,57]. Overall, this combination of moderate interaction strength and geometrical specificity makes π–π stacking a consistent and relevant adsorption mechanism in aqueous systems.

Three-dimensional reduced graphene oxide aerogels show high PS adsorption capacities (up to 617.28 mg g^−1^), likely due to the combined contribution of π–π interactions with the sp^2^ carbon framework and pore-filling within their porous structure [58,59]. For biochar, the aromaticity of the condensed sp^2^ domains, and consequently the π-stacking tendency, generally increases with pyrolysis temperature, providing a tunable synthetic lever to enhance PS uptake [31,60]. Because π–π interactions are generally less sensitive to changes in ionic strength and pH compared to electrostatic interactions, they often represent a relatively stable contribution in complex aqueous matrices.

### 3.3. Electrostatic Interactions

Electrostatic forces emerge as a complementary and often controlling mechanism when charged surfaces are involved. Under environmentally relevant conditions (pH 4–8), MNPs typically exhibit a slightly negative ζ-potential, particularly after environmental aging, due to ion adsorption and surface oxidation processes. As a result, adsorbents bearing positively charged functionalities, such as protonated amines, brucite-layer metal hydroxides, and cationic metal–organic frameworks, experience Coulombic attraction toward these particles [3,4,26].

The ability to tune surface charge is equally important from a materials-design perspective. Bio-based substrates such as chitin and cellulose can be chemically modified to introduce pH-responsive or permanently charged functional groups, enabling selective adsorption of specific MPs depending on their surface chemistry and environmental conditions [61].

Electrostatic attraction plays a major role in several high-performing adsorbents. For instance, Zn/Al layered double hydroxides (LDHs), characterized by intrinsically positively charged brucite-like layers, exhibit adsorption capacities of 162.62 and 53.0 mg g^−1^ toward PS nanoplastics in synthetic freshwater and hard water, respectively, primarily driven by Coulombic interactions [62]. Similarly, the cobalt-based framework ZIF-67 achieves removal efficiencies of 92.1% for PS MPs, where electrostatic attraction operates in conjunction with π–π stacking and hydrogen bonding [63]. In this system, adsorption efficiency is maintained across a broad pH range (3–10), but decreases markedly at higher pH, where surface charge neutralization and increasing electrostatic repulsion reduce affinity.

Compared to other interaction modes, electrostatic forces are highly sensitive to solution chemistry. Variations in pH directly affect the surface charge of both MPs and adsorbents, while ionic strength can influence the thickness of the electrical double layer and the effective interaction distance. In particular, divalent cations such as Ca^2+^ and Mg^2+^ can mediate cation-bridging interactions between similarly charged surfaces, effectively overcoming electrostatic repulsion and promoting aggregation or adsorption. This effect helps explain the unexpectedly high performance of some adsorbents in contaminated-water systems [64].

Overall, electrostatic interactions represent one of the most tunable mechanisms in MP adsorption systems, enabling selectivity through both material design and environmental control. However, their dependence on pH and ionic composition also makes them less robust than π–π or hydrophobic interactions under complex real-water conditions.

### 3.4. Hydrogen Bonding

Building on the interaction mechanisms discussed above, hydrogen bonding represents a complementary adsorption pathway that becomes increasingly relevant as the surface chemistry of MNPs evolves under environmental conditions. Hydrophobic interactions predominantly govern the adsorption of nonpolar polymers, whereas electrostatic forces control charge-driven processes. In contrast, hydrogen bonding generally provides a secondary but non-negligible contribution, becoming significant when polar functional groups are present on either the polymer surface or the adsorbent, particularly in aged or functionalised plastics [36,38,65].

This interaction becomes particularly relevant for environmentally aged MPs and NPs, whose surfaces are progressively enriched in oxygen containing functional groups such as hydroxyl, carbonyl, and carboxyl groups as a result of oxidative weathering and biofouling, leading to increased surface polarity [66,67]. These groups act as hydrogen bond donors or acceptors, resulting in interactions with adsorbents bearing complementary functionalities [8,68]. On the adsorbent side, materials rich in oxygen and nitrogen functionalities, such as oxidized biochars, cellulose-based substrates, chitin, chitosan, and amine-functionalized polymers, provide abundant OH, NH_2_, and COOH groups that can readily form hydrogen bonds with oxidized polymer surfaces [69,70,71].

Compared to electrostatic interactions, hydrogen bonding is generally less sensitive to ionic strength, as it does not rely on long range Coulombic forces. However, its strength and contribution can still be influenced by pH, which governs protonation and deprotonation equilibria and thereby affects the availability of donor and acceptor sites on both MPs and adsorbent surfaces. As a result, hydrogen bonding is typically favoured under near neutral conditions, whereas strongly acidic or alkaline environments may reduce its contribution by altering surface speciation [72]. Experimental evidence for hydrogen bonding in MP adsorption systems is often obtained through spectroscopic analysis. In particular, FTIR measurements may show shifts and broadening of O–H stretching bands after adsorption, which can be indicative of intermolecular hydrogen bonding between the adsorbent and the plastic surface. Such features have been reported, for instance, in cellulose-based adsorbents interacting with functionalized nanoplastics, where hydrogen bonding is considered to contribute to enhanced affinity toward more polar particles [61,73].

In practical systems, hydrogen bonding rarely acts alone and typically occurs alongside other interaction mechanisms. For example, composite materials such as chitin-based or amine-functionalized aerogels combine hydrogen bonding with electrostatic interactions and π–π stacking, enabling efficient removal across different polymer types [20,71].

Overall, hydrogen bonding provides a moderate but selective contribution, which may become more relevant under environmentally realistic conditions where plastic surfaces are increasingly oxidized and heterogeneous.

### 3.5. Pore Filling

Whereas the mechanisms discussed above are largely governed by surface chemistry, pore filling can be described as a complementary process primarily controlled by the structural correspondence between the pore architecture of the adsorbent and the size of the target particles. In highly porous materials such as aerogels, hierarchical carbons, MOFs, and covalent organic frameworks (COFs), physical confinement of plastic particles within the pore network can contribute to adsorption and is often associated with intraparticle diffusion processes [4,20,74]. Diffusion within the pore network may also influence the overall uptake rate, particularly at higher concentrations [3,38].

Adsorption kinetics in porous materials are often governed by intraparticle diffusion and mass transfer, and can be reasonably described by simplified models that link equilibrium behaviour with uptake rate [39,75]. Effective pore filling requires pore dimensions that allow plastic particle access, although the optimal pore to particle size ratio also depends on factors such as pore connectivity and tortuosity [20]. Once encapsulated, particles can interact with the internal surface via additional mechanisms, such as van der Waals forces, electrostatic attraction, and hydrophobic interactions, over an increased contact area, thereby enhancing overall adsorption capacity. Yuan et al. [59] successfully employed graphene-based aerogels, showing that appropriately designed pore structures can enable efficient capture of MPs. Conversely, the contribution of pore filling tends to decrease when there is a mismatch between pore size and particle size. Large pores provide limited confinement, while particles larger than the pore openings may be excluded. This size dependence has been reported in porous systems, where retention mechanisms can shift from pore related adsorption to straining, surface interactions, or aggregation as particle size increases [76,77,78].

Overall, pore filling can be considered as a size-dependent and complementary mechanism, whose contribution increases when the pore size distribution is well matched to the particle size. In this context, materials with hierarchical porosity are often more effective, as they can accommodate a broader range of particle sizes and adsorption pathways.

### 3.6. Chemisorption and Surface Complexation

Chemisorption involves the formation of chemical bonds between the adsorbate and the adsorbent surface, governed by surface chemistry and, in particular, by the presence of reactive functional groups on the adsorbent [3]. Within this framework, surface complexation can be regarded as a specific manifestation of chemisorption, whereby coordination bonds form between surface hydroxyl or metal–oxygen sites and oxidised moieties present on MNPs.

Although distinguishing chemisorption from strong physisorption through macroscopic isotherms has long been challenging, molecular-level evidence has now clarified its role. Density functional theory (DFT) calculations by Li et al. [26] show that Fe-doped magnetic sponge carbon forms Fe–O–polymer bonds with PS, increasing adsorption energies beyond the physisorption regime and enabling rapid uptake (369.07 mg g^−1^ within 10 min). Consistent evidence is provided by XPS measurements, which reveal C 1s binding-energy shifts and changes in oxygen-containing functional groups after NP adsorption, indicative of ionic and covalent bonding. FTIR analysis further suggests that hydrogen bonding may coexist with these interactions, indicating that surface complexation typically operates alongside weaker mechanisms rather than in isolation [3].

Together, these findings establish a consistent framework in which bond formation governs adsorption in metal-functionalised and oxygen-rich materials. This behaviour becomes more pronounced at smaller particle sizes. Due to their higher specific surface area, NPs expose a greater density of reactive sites and are therefore more prone to chemisorption. Accordingly, adsorption kinetics of NPs across a wide range of materials, including MXenes and metal-modified carbons, are often well described by pseudo-second-order models, suggesting that chemisorption may play an important role in the rate-controlling step [79,80].

Within this chemisorption regime, surface complexation is an important pathway in high-performance adsorbents. Wu et al. [80] employed magnetic biochar (MRB), prepared from agricultural waste rice husks, to efficiently adsorb MPs in aquatic systems (99.96% of MP removal). The results indicated that surface complexation contributes to adsorption by forming direct interactions between MPs and oxygen- and iron-containing functional groups on the biochar surface, as confirmed by FTIR and XPS shifts indicating changes in chemical bonding. Similar results were observed for co-pyrolysed biochars removing PS sulfonate, where exothermic and spontaneous adsorption occurs and surface complexation contributes by enabling interactions between MPs and functional groups on Fe-modified biochar surfaces [79]. Comparable mechanisms occur in inorganic adsorbents such as Zn/Al layered double hydroxides, where charged layers and hydroxyl groups favour coordination with oxidised polymers, especially when pore access is limited [4,62].

Across these materials, kinetics usually follow pseudo second order behaviour, consistent with, rather than conclusive proof of, electron sharing or transfer as the rate limiting step [79,80,81].

**Figure 1:**
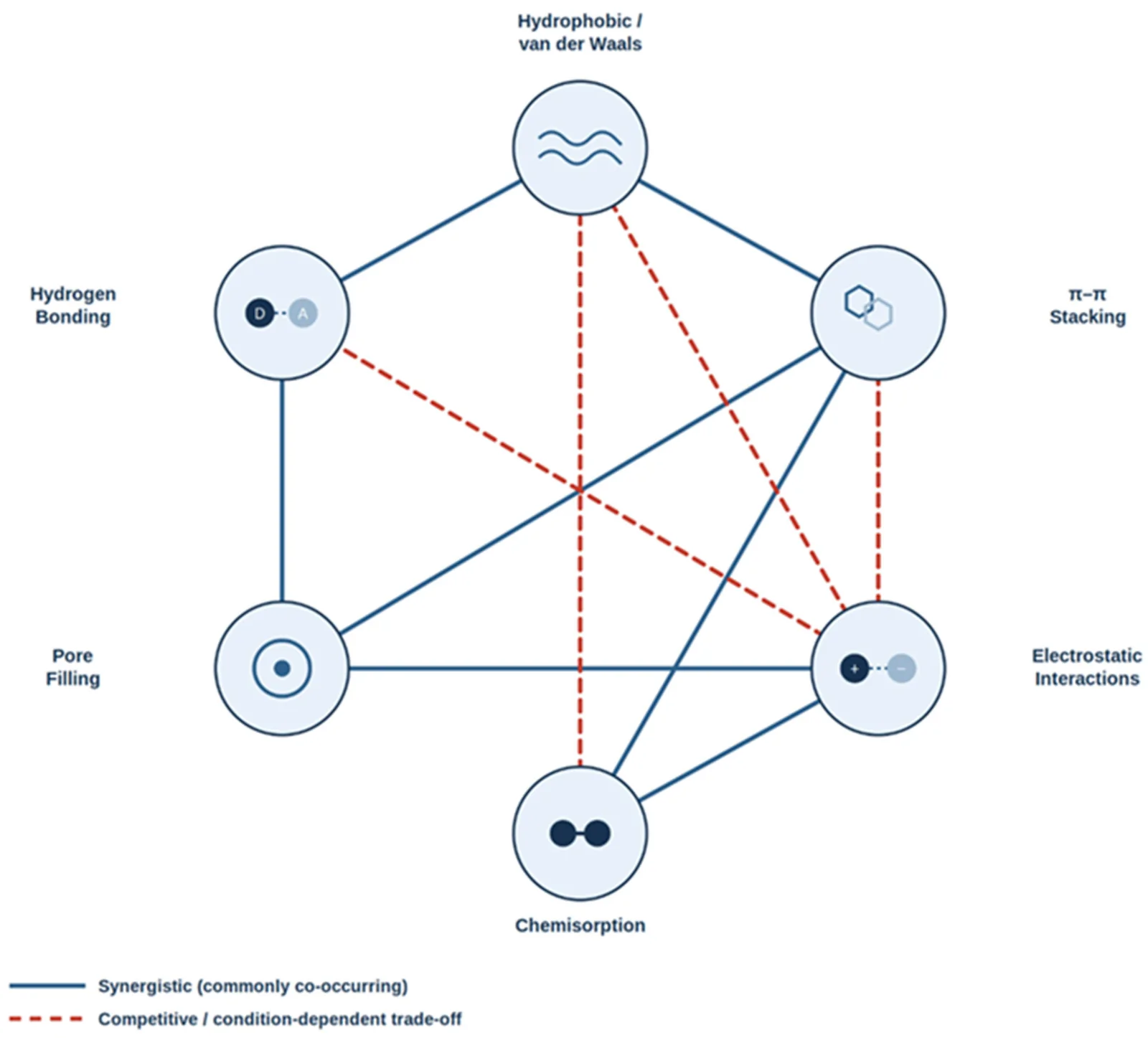
Relationships among the six molecular interaction mechanisms. Solid lines indicate mechanisms that commonly co-occur, whereas dashed lines indicate potential competition or trade-offs depending on solution conditions and surface properties.

Overall, adsorption arises from the combined action of multiple mechanisms, influenced by particle size, surface chemistry and structure. Chemisorption becomes more dominant at the nanoscale, guiding the design of adsorbents with abundant reactive groups. These interactions do not operate in a purely additive manner. As summarised in Figure 1, several mechanisms often occur simultaneously and may complement one another, such as hydrophobic interactions and π–π stacking on carbon-rich surfaces, or pore filling together with electrostatic attraction and hydrogen bonding in porous materials. In contrast, the contribution of others may vary depending on solution conditions and surface chemistry, potentially leading to competing effects or trade-offs. Electrostatic attraction is comparatively sensitive to ionic strength and pH, and its contribution can weaken or reverse under high salinity or unfavourable pH, while hydrophobic and π–π interactions remain relatively stable under the same conditions [64]. A related trade-off applies to hydrogen bonding: on amine-bearing adsorbents, protonation promotes electrostatic attraction toward MNPs [61], and the same protonation is expected to reduce the availability of these groups as hydrogen bond donors or acceptors, a pattern also described in the broader adsorption literature [82]. Chemisorption can similarly compete with weaker physisorptive pathways for a finite number of high-affinity surface sites, a site-heterogeneity effect documented for other contaminant classes on carbon-based adsorbents and expected to extend to MNP systems, where high-affinity sites are preferentially occupied first, leaving comparatively less reactive sites available for hydrophobic or van der Waals contributions [83]. As a result, the dominant pathway for a given adsorbent-polymer pair can shift with water chemistry and surface functionality rather than remaining fixed.

## 4. Adsorbents for MNP Removal

The mechanistic framework outlined in the previous section suggests that adsorption performance is not governed by a single factor, but rather emerges from the combined action of multiple interaction modes. The relative importance of these interactions varies depending on both the structure of the adsorbent and the surface chemistry of the plastic. Building on this perspective, the following paragraphs examine five material classes frequently discussed in recent studies, including carbon-based adsorbents, bio-based and waste-derived materials, porous engineered scaffolds, MOFs and COFs, and metal (hydr)oxides with magnetic composites. The aim is to highlight the key structural and chemical features of each material class, while noting considerations related to scalability and sustainability. Given the wide variation in experimental conditions across studies, the adsorption capacities reported below should be viewed as indicative of potential rather than directly comparable performance metrics.

### 4.1. Carbon-Based Adsorbents

Carbon-based materials, such as graphene, carbon nanotubes, biochar, and activated carbon, constitute a broad and highly promising class of adsorbents for MNP removal, owing to their structural tunability and consistently high adsorption performance [3,28,31,71,84]. Their effectiveness derives from key physicochemical properties, including high specific surface area, hierarchical porosity, and chemically versatile surfaces, combined with the possibility of synthesis from both fossil precursors and renewable biomass, which supports scalability [85]. By modulating surface chemistry and pore structure, the same carbon material scaffold can promote multiple interaction pathways, including hydrophobic interactions, π–π stacking, hydrogen bonding, and electrostatic attraction, which are widely recognized as the dominant mechanisms governing MNP adsorption [31,86]. This versatility establishes carbon adsorbents as benchmark materials for comparison with emerging technologies.

Biochar represents the most accessible and cost-effective entry within this family, as it can be readily produced from agro-forestry residues such as rice husk, corncob, coconut husk, and other lignocellulosic feedstocks [87,88,89]. Although biochar derives from biomass, it is here classified as a carbon-based adsorbent because pyrolysis converts the precursor into a carbonaceous structure whose adsorption is governed by aromatic domains, microporosity, and tunable surface chemistry, with properties largely controlled by the pyrolysis temperature. High-temperature biochars are enriched in condensed sp^2^ structures that favour π–π interactions with aromatic polymers such as PS, whereas lower-temperature materials retain hydroxyl and carboxyl groups that enhance hydrogen bonding with oxidised plastics [4,31]. This tunability can be further improved by post-treatment, for example through oxidation, which increases surface polarity and enhances adsorption of PS nanoplastics [90]. Application studies confirm this behaviour. Siipola et al. [87] showed that pine and spruce bark biochar effectively retains large particles, while removal of micrometre-scale MPs is less efficient, indicating size-dependent retention. Similarly, Wang et al. [91] reported over 95% removal of 10 µm PS microspheres using corn straw and hardwood biochars, outperforming sand filtration. Microscopy revealed retention through interparticle spaces, pore trapping, and entanglement by detached biochar fragments, highlighting the combined role of structural confinement and weak hydrophobic and van der Waals interactions in MP removal.

Activated carbon (AC), in both granular and powdered forms, represents a higher-performance counterpart to biochar, owing to its more extensively developed microporous network. Arenas et al. [84] investigated the removal of positively charged PS nanoplastics (~100 nm) using granular activated carbon in drinking-water matrices. In natural surface water from Lake Geneva, used as a realistic drinking water matrix, the capture mechanism was strongly influenced by water chemistry. Adsorption was promoted by aggregation processes and interactions with dissolved organic matter, while electrostatic interactions remained important. Divalent cations such as Ca^2+^ and Mg^2+^ further enhanced removal by acting as bridges between negatively charged species and the carbon surface. Under these conditions, removal efficiencies reached up to about 90%, with adsorption capacities up to 6.33 mg g^−1^. More recently, Laca et al. [92] evaluated the removal of PP microplastics (20–500 μm) by granular activated carbon in wastewater-related matrices. When treating synthetic suspensions and real wastewater samples, removal efficiencies ranged from about 30–43% under typical conditions (0.5 g L^−1^ GAC, 7 h), increasing up to ~90% at higher adsorbent dosages (1.5 g L^−1^). Hybrid surface engineering can lift this baseline further: the KOH-activated, chitosan-coated anthracite developed by Lv et al. [22] achieves substantially improved removal of PVC microplastics through a synergy of physical interception, electrostatic attraction, and polymer-mediated bridging—effectively stacking multiple interaction modes within a single material architecture.

Across carbon-based adsorbents, a general trend can be observed, with hydrophobic interactions and π–π stacking providing the basic adsorption mechanism. This behaviour can be gradually enhanced through surface modifications such as oxidation, heteroatom doping, and iron incorporation, which introduce additional interactions including hydrogen bonding, electrostatic effects, and chemisorption. Overall, carbon materials can be designed by tuning their surface properties to favour the interaction pathway most relevant to the target polymer and application context [3,4,31].

### 4.2. Bio-Based and Biowaste Adsorbents

Bio-based and biowaste-derived adsorbents represent a class of sustainable materials that extend the logic of biomass valorisation by exploiting natural polymers and minimally processed residues rather than fully carbonised structures. These systems retain a substantial fraction of their original chemistry, including celluloses, lignins, chitins, and related polysaccharides, and can often be obtained through low-temperature or mechanical processing routes. Their growing relevance stems from the fact that natural polymers and their composites are renewable, frequently biodegradable, and structurally tunable, which allows their surface functionality and morphology to be tailored to specific adsorption targets [93,94,95].

From a mechanistic perspective, the abundance of polar functional groups such as hydroxyl, carboxyl, and amino moieties provides intrinsic adsorption sites, enabling interactions such as hydrogen bonding, electrostatic attraction, and physical entrapment alongside hydrophobic effects. This built-in chemical versatility reduces the need for aggressive activation steps while supporting multiple interaction pathways with microplastics and other contaminants [96]. These materials combine low cost, abundant renewable feedstocks, and low environmental impact, but show more variable performance and limited regeneration compared to engineered carbons, often leading to single-use applications [8]. Typical examples include cellulose- and chitosan-based materials, lignin adsorbents, alginate or pectin gels, agricultural residues, and hybrid biopolymer composites, spanning architectures from raw biomass to structurally tuned materials [97,98].

A direct and practical implementation of this concept is represented by the use of minimally processed agro-industrial residues as low-cost bio-based adsorbents. Alnasrawy [99] showed that mechanically processed banana peels, dried at 60 °C, capture more than 99% of MPs from synthetic solutions and 97.5% from real WWTP effluent under optimal conditions (pH 4, 300 mg L^−1^ dose, 25-min contact time). The lignocellulosic matrix provides hydroxyl, carboxyl, and phenolic groups that drive electrostatic attraction and hydrogen bonding toward oxidised MP surfaces. Comparable performance has been reported for sugarcane bagasse, coconut bagasse, and coffee grounds, all of which exploit the hydroxyl-rich chemistry of their cellulose backbone [8].

Surface modification can enhance the performance of low-cost adsorbents without requiring thermal activation. Oliveira et al. [100] employed Tween-80-based microemulsions to functionalise materials such as sugarcane and coconut bagasse, banana-peel residue, bentonite, and diatomite, improving their affinity toward microplastics. The modified systems achieved removal efficiencies of up to ~80% for mixed microplastic suspensions, likely due to enhanced hydrophobic interactions at the adsorbent–particle interface. This approach demonstrates how introducing amphiphilic character can compensate for the limited intrinsic non-polarity of biowaste substrates, although the use of surfactants also raises considerations regarding their environmental fate.

Moving from raw agro-industrial residues to more refined biopolymeric substrates, cellulose-based adsorbents offer a complementary route in which surface charge can be introduced with greater precision through chemical functionalisation. Batool and Valiyaveettil [61] showed that polyethyleneimine-modified cellulose fibers can achieve very high removal efficiencies for functionalised PMMA, poly(vinyl acetate) (PVAc), and PVC nanoplastics, largely due to the presence of protonated amine groups that impart a positive surface charge and promote electrostatic attraction toward negatively charged particles.

Collectively, hydrogen bonding and electrostatic interactions appear to be the main and most readily adjustable mechanisms, arising from the native surface chemistry of these materials. Additional modifications, such as surfactant or amine functionalisation, can further support hydrophobic and Coulombic contributions, leading to improved performance. Overall, this approach allows for enhanced adsorption while still maintaining the advantages of renewable feedstocks and relatively low-energy processing.

### 4.3. Porous Engineered Materials: Sponges, Aerogels, Hydrogels

Owing to their high porosity and large accessible surface area, sponge-, aerogel-, and hydrogel-based materials enable efficient removal of MNPs through a combination of physical confinement and surface-mediated interactions. The adsorption process generally involves three sequential stages, namely transport from the bulk solution to the external surface, diffusion within the porous network, and the establishment of equilibrium between retained and suspended particles [101,102,103]. Unlike carbon- and bio-based adsorbents, where surface chemistry plays a dominant role, these systems also strongly rely on pore filling and intraparticle diffusion as key capture mechanisms [20].

Their main advantages include very high porosity, often exceeding ninety percent by volume, low density, and highly tunable surface chemistry. However, they often suffer from limited mechanical stability under repeated use and generally lack established routes for scale-up beyond laboratory batch systems.

Within this framework, biopolymer-based sponges represent a particularly sustainable class of materials. A chitin–graphene oxide (ChGO) sponge demonstrated removal efficiencies up to 89.8% for PS, 72.4% for carboxylated PS, and 88.9% for aminated PS in synthetic wastewater. These performances arise from the combined effect of electrostatic interactions, hydrogen bonding, and π–π interactions, which act synergistically with pore confinement and diffusion processes. Variations in removal efficiency reflect differences in MP surface functionalization, which directly influence electrostatic interactions and adsorption affinity toward the ChGO matrix [71]. Similarly, plant protein-based sponges provide a cost-effective and sustainable alternative. An oat protein sponge achieved removal efficiencies up to ~81% for PS microparticles (1 μm), benefiting from a highly porous interconnected structure (~83%) and rapid adsorption kinetics. In this case, adsorption is mainly governed by hydrophobic interactions between protein side chains and the aromatic structure of polystyrene, combined with intraparticle diffusion within the sponge network. These mechanisms enable fast uptake while maintaining reusability and biodegradability [104].

More recently, aerogels have emerged as highly promising platforms for MNP removal due to their low density, high specific surface area, and interconnected three-dimensional porous structure [105]. Their performance stems from the combination of hierarchical porosity and tunable surface chemistry, enabling multiple adsorption mechanisms such as pore filling, electrostatic interactions, hydrogen bonding, hydrophobic interactions, and π–π stacking. Cellulose- and polysaccharide-based aerogels typically achieve removal efficiencies above 90% and adsorption capacities ranging from ~100 to over 500 mg g^−1^, depending on pore structure and functionalization [20]. For instance, D-DPGG aerogels reach ~241 mg g^−1^ with ~96% PS removal [106], while superhydrophobic composites can exceed 550 mg g^−1^ and 95% efficiency for PS nanoplastics (50 nm) [107]. Biomass-derived systems such as gelatin–sodium alginate aerogels offer a particularly sustainable alternative, maintaining removal efficiencies above 90% across a wide pH range and in different water matrices. In these materials, adsorption arises from a synergistic combination of physical entrapment and surface interactions, particularly hydrogen bonding and ionic interactions [20]. Polydopamine-modified magnetic chitosan aerogels (PDA-MCS) further highlight the practical applicability of this class. Zheng et al. reported a 91.6% removal of PET MPs at pH 6–9 together with magnetic recoverability, resulting from the combined effects of electrostatic and hydrogen-bonding interactions provided by chitosan, the hydrophobic affinity introduced by the polydopamine coating, and the ease of separation under an external magnetic field [74]. These materials also showed stable performance under simulated environmental conditions, achieving removal efficiencies of 94.6% for PE and 92.3% for PS microplastics.

Hydrogels complement this class by introducing structural flexibility and tunable surface properties. For example, a polydopamine-modified sodium alginate hydrogel [74] achieved ~99.6% microplastic removal, where polydopamine enhances hydrophobicity and overall surface affinity. Similarly, fully bio-based chitin–lignin hydrogels [108] reached very high adsorption capacities (up to 1790.8 mg g^−1^ for PS nanoplastics), mainly driven by synergistic electrostatic attraction and π–π interactions. These systems also exhibit good regeneration potential and scalable synthesis routes.

Overall, a clear relationship between structure and performance emerges across these materials. While pore filling represents the primary mechanism, it is typically enhanced by surface interactions such as π–π stacking, electrostatic attraction, and hydrogen bonding. At the same time, adsorption efficiency depends on an appropriate match between pore size and particle dimensions, as excessively large pores can reduce retention. As a result, current research is increasingly focused on improving mechanical stability and achieving precise control over pore architecture to support practical applications.

### 4.4. Metal–Organic and Covalent-Organic Frameworks (MOFs/COFs)

Metal–organic frameworks (MOFs) and covalent organic frameworks (COFs) introduce highly ordered, crystalline porosity at the atomic scale into the field of MNP adsorption, representing one of the most structurally and chemically sophisticated classes of materials among those considered. Their tunability, achieved through the choice of metal nodes, organic linkers, pore size, and post-synthetic modification, allows precise control over surface charge, hydrophobicity, and chemical reactivity, enabling mechanism-selective design that is difficult to achieve in amorphous adsorbents [109,110,111].

Reported removal efficiencies typically range from 70 to 99.9%, with performance influenced by particle characteristics, water chemistry, and framework structure. Smaller nanoplastics are often removed more effectively than larger microplastics due to their higher surface area and stronger interfacial interactions. The main limitations of this material family include complex synthesis routes, variable stability in aqueous environments, and the largely unresolved environmental implications of potential metal leaching during regeneration [4,20].

Among MOFs, ZIF-67 has become a widely studied system for polystyrene microplastic capture. Wan et al. [63] reported a removal efficiency of 92.1% for PS MPs smaller than 10 μm at a dosage of 5 mg mL^−1^ over a pH range from 3 to 10. The process was mainly driven by electrostatic attraction between the positively charged framework and the negatively charged PS surface, supported by π–π interactions between the aromatic linker and polymer chains and by hydrogen bonding mediated by coordinated water molecules. At pH values above 10, both surfaces become negatively charged, leading to a sharp decline in performance and highlighting the dependence of charge-driven adsorption on solution conditions. A broader survey of MOF performance across polymer types confirms the generality of this mechanistic picture [111]. Modak et al. [112] showed that Cr-MOF/MIL-101 reaches 96% removal of PS nanoplastics at pH 5, with an adsorption capacity of 800 mg g^−1^, governed by electrostatic attraction, π–π interactions, and acid–base interactions, and following pseudo-first-order kinetics together with a Freundlich isotherm consistent with multilayer adsorption on a heterogeneous surface. Liu et al. [113] reported that Fe_3_O_4_@SiO_2_@MIL-53(Al) achieves removal efficiencies of 93.17% for PVC, 85.16% for PP, and 88.28% for polyester (PES) from mixed-polymer suspensions, with pseudo-second-order kinetics and Langmuir isotherms, both of which are often interpreted as indications of chemisorption involvement, although they do not constitute direct evidence that chemisorption is the rate-controlling step; the reported removal efficiencies further suggest that magnetic functionalisation does not compromise polymer-type selectivity. Yet, Gnanasekaran et al. [114] demonstrated that a PSF/MIL-100(Fe) membrane achieves removal efficiencies above 99% for PS nanoplastics from textile wastewater, maintaining its performance over six regeneration cycles due to the stable electrostatic and π–π interaction sites provided by the organic ligands within the framework, representing one of the few examples of multi-cycle stability under realistic effluent conditions.

Magnetic functionalisation offers a practical solution to material recovery without compromising performance. Feng et al. [115] synthesised a series of Fe_3_O_4_@carboxymethylcellulose(CMC)-MOF composites and identified Fe_3_O_4_@CMC-MIL-101-NH_2_ as the optimal system, achieving 98.0% removal and an adsorption capacity of 245.1 mg g^−1^ for PS MPs (3 µm) within 240 min, with a theoretical Langmuir maximum of 1923 mg g^−1^ and retention of above 89% efficiency after five regeneration cycles. DFT calculations and XPS analysis attributed the adsorption to van der Waals interactions between PS hydrocarbon chains and the Fe clusters and carboxylate groups of MIL-101-NH_2_, complemented by π–π stacking between the PS aromatic rings and the amino-functionalised linker. Broad-spectrum validation showed capacities exceeding 250 mg g^−1^ across PP, PE, PMMA, PVC, and PET particles of varying size, shape, and surface charge, while removal efficiencies above 81% were maintained in complex beverage matrices including carbonated drinks and sports beverages.

Beyond pure adsorption, MOF-based composites are beginning to demonstrate integrated capture-and-degrade functionality. Zanaty et al. [116] synthesised an HTNT@ZIF-67 nanocomposite by in-situ growth of ZIF-67 crystals onto hydrogen titanate nanotubes at room temperature, and applied it to MPs extracted from commercial toothpaste, representing a realistic, polymer-heterogeneous target comprising PE, PP, PET, and PMMA fragments. The composite achieved 97% removal efficiency through a sequential adsorption–catalytic oxidation pathway in which the porous ZIF-67 framework first concentrates MP particles via electrostatic attraction and π–π interactions, after which H_2_O_2_ activation by the cobalt nodes drives Fenton-like oxidative chain scission, confirmed by FTIR disappearance of aromatic C=C stretching at 1630 cm^−1^ and a marked reduction in particle dimensions by optical microscopy, with the catalyst retaining 78% efficiency after six cycles and showing negligible cobalt leaching.

COFs represent a structurally distinct class compared to MOFs. Built entirely from covalent organic linkages, they form highly stable, π-conjugated networks without metal centers, which favour the adsorption of aromatic MPs such as polystyrene through strong π–π interactions. In contrast, aliphatic polymers such as PE and PP interact mainly via hydrophobic forces, leading to a degree of selectivity that is difficult to achieve with conventional MOFs [4]. AlNeyadi et al. [117] developed Fe_3_O_4_@MP-COF/PVDF composite membranes combining adsorption and visible-light photocatalysis. The system achieved adsorption capacities up to 520 mg g^−1^ for PS, 380 mg g^−1^ for PP, and 280 mg g^−1^ for PE, demonstrating faster uptake and higher selectivity compared to non-aromatic polymers. Under visible light, the Fe_3_O_4_–COF interface promotes reactive oxygen species formation, enabling up to 98% degradation of PS and significant TOC reduction, while maintaining good stability over repeated cycles. This dual functionality offers a promising strategy to couple microplastic capture with in situ degradation, reducing the risk of secondary release. Li et al. [118] synthesized a cationic HTA-EB iCOF and integrated it with chitosan and tannic acid to produce a lightweight macroporous aerogel with a density of 33 mg cm^−3^. This system combines electrostatic, π–π, and hydrogen bonding interactions with a three-dimensional elastic structure, achieving adsorption capacities of 550.73 mg g^−1^ for PET, 341.74 mg g^−1^ for PS, and 209.32 mg g^−1^ for PC, with removal efficiencies above 70% in real water matrices. The aerogel architecture also addresses key limitations of powder COFs such as aggregation, difficult recovery, and potential secondary release, while introducing antibacterial and antifouling properties that support long-term operation.

Aluminosilicates such as zeolites, molecular sieves, and bentonite-based composites provide an earth-abundant and scalable alternative to crystalline frameworks at significantly lower cost [119]. Shen et al. [120] showed that granular zeolite and molecular sieve materials modified with a cationic surfactant (1-hexadecylpyridinium bromide—HDPB) removed more than 96% of PE and PA MPs from synthetic wastewater, outperforming conventional rapid sand filtration. Microscopic analysis revealed that adsorption occurs through particle capture, trapping, and entanglement within the porous structure, consistent with pore-filling and confinement mechanisms. This approach has also been validated at pilot scale. Spacilova et al. [21] reported that a modified zeolite and bentonite system operating in a developed pilot plant sorption maintained MP removal efficiencies above 90% over one year, representing one of the few long-term field demonstrations available.

Overall, the high degree of structural control offered by crystalline frameworks makes them particularly suitable for rational adsorbent design, whereas aluminosilicates represent the most cost-effective and viable option for large-scale implementation, bridging the gap between laboratory performance and real wastewater treatment applications.

### 4.5. Metal (Hydr)oxides and Magnetic Composites

Metal (hydr)oxides and magnetic composites are distinguished by their ability to couple chemisorption, arising from coordination or covalent interactions at metal oxygen surface sites with oxidised MNP functional groups, with magnetic separability, thereby enabling the recovery and reuse of fine particulate adsorbents in continuous treatment systems [26,121]. This dual functionality distinguishes them from purely physisorptive adsorbents, where pseudo second order kinetics often reflect diffusion limited uptake rather than true surface bonding.

Layered double hydroxides represent the most widely studied subfamily. Tiwari et al. [62] synthesized a Zn-Al layered double hydroxide adsorbent to remove hydrophilic PS nanoparticles coated with an anionic surfactant from synthetic freshwater. The results demonstrated adsorption capacities up to 162.62 mg g^−1^ for PS nanoplastics, driven by electrostatic attraction between positively charged layers and negatively charged particles, reinforced by surface complexation via hydroxyl groups. Yang et al. [122] developed a superparamagnetic Mg–Al layered double oxide loaded with Fe_3_O_4_ (M-MgAl-LDO) for PS microplastic removal, achieving capacities up to 203.12 mg g^−1^ at 25 °C, outperforming most of the existing adsorbents. Adsorption followed pseudo-second-order kinetics and a Langmuir isotherm, patterns consistent with the possible involvement of monolayer adsorption and chemisorption, although such model fits alone cannot be considered definitive mechanistic evidence; additional diffusion limitations emerged at higher concentrations. Mechanistically, uptake involved pore filling, π–π interactions, electrostatic attraction, hydrogen bonding, and metal-oxide complexation, while magnetic recovery highlights its practical applicability.

Within this framework, iron-based materials emerge as a particularly prominent subclass, where the chemisorptive character of metal (hydr)oxides is further enhanced by the redox activity and strong surface reactivity of Fe species. Singh et al. [121] prepared Fe_3_O_4_-modified biochars pyrolyzed at two different temperatures, i.e., 550 °C (FB-550) and 850 °C (FB-850), from *Prosopis juliflora*. The adsorption performance was evaluated using PS nanoplastics with varying sizes (30–1000 nm) and surface functionalities. Both materials exhibited rapid removal (<10 min), high adsorption capacities (up to 290.20 mg g^−1^), and good reusability without significant iron leaching. Kinetic analysis indicated fast, reaction-controlled uptake, while spectroscopic evidence confirmed inner-sphere complexation between NP functional groups and iron hydroxyl sites. Electrostatic interactions and biochar surface properties further modulated adsorption selectivity. Similar behaviour was observed by Li et al. [26], who developed a Fe-doped magnetic sponge carbon (FeMSC) with a high surface area and strong magnetic properties via one-step pyrolysis. The material showed rapid adsorption of PS microplastics (saturation <10 min) with a high capacity (369.07 mg g^−1^) and stable performance across varying pH and real water matrices. Kinetics and DFT analysis confirmed chemisorption, with Fe sites promoting strong Fe–C coordination alongside enhanced π–π interactions. Extending this approach, magnetic biochar derived from rice straw achieves up to 99.96% removal and retains about 90% capacity after four regeneration cycles [80]. Zhao et al. [123] employed Fe-modified fly ash for PS NP removal, achieving adsorption capacities of 82.8–89.9 mg g^−1^ with good reusability. Mechanistic analysis indicated that electrostatic attraction, surface complexation, and π–π interactions jointly governed the adsorption process.

Recent developments expand both structural design and feedstock diversity. Kim et al. [79] produced a magnetic biochar via co-pyrolysis of walnut shells and Fe-rich mine tailings, achieving PS-sulfonate capacities of 0.77–6.75 mg g^−1^ across a molecular mass range of 32,000–210 Da. Interestingly, the pronounced size-dependent trend reflected a mechanistic shift from hydrophobic and π–π interactions at larger sizes toward Fe-mediated surface complexation and electrostatic attraction as particle dimensions decrease toward the nanoscale. Li and co-workers [124] pursued a structural innovation by grafting N-octadecylphosphonic acid onto amino functionalised Fe_3_O_4_@SiO_2_ cores, producing amphiphilic Janus microparticles. The hydrophobic domain captures PE through van der Waals interactions, while the positively charged domain removes PS via electrostatic attraction and cation–π interactions, achieving 92% PS and 61% PE removal within 20 min with full magnetic recoverability. To our knowledge, this represents the only adsorbent design explicitly tailored to target both polar and non polar polymers within a single particle.

Across this adsorbent class, magnetic recoverability represents both a defining advantage and a practical limitation, as repeated cycling can induce oxidation, partial loss of crystallinity, and metal leaching into treated water. Overall, metal hydroxides and magnetic composites can promote the full spectrum of interaction mechanisms described above, from electrostatic attraction and hydrogen bonding to chemisorption at metal oxygen sites. The increasing use of low cost waste-derived supports such as biochar, fly ash, and mining residues further links adsorption performance to circular feedstocks, positioning this family as a bridge between high efficiency capture and scalable sustainable remediation.

**Figure 2:**
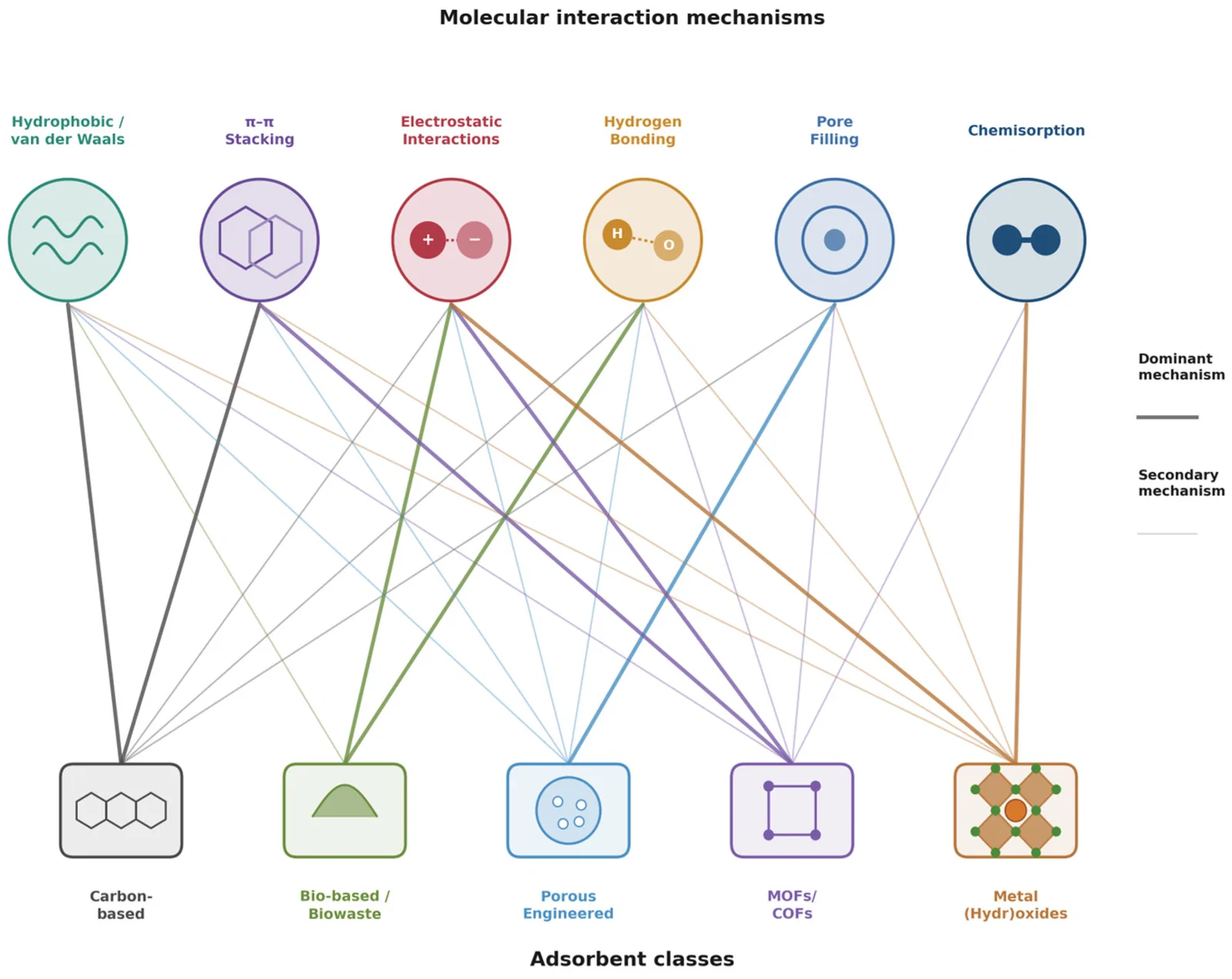
Dominant and secondary molecular interaction mechanisms mapped across the five adsorbent classes discussed in this review.

The interaction pathways discussed across these five adsorbent classes are summarised graphically in Figure 2, which maps the dominant and secondary molecular mechanisms identified for each material family. A mechanism is classified as dominant when this section identifies it as the primary driver of adsorption for a given class, based on the weight of evidence reported across the cited studies, and as secondary when it is described as contributing to or modulating performance without being identified as primary. This criterion better clarifies the role of π–π stacking, shown as dominant mainly where aromatic structures are involved, such as carbon-based materials and MOFs/COFs interacting with PS or PET, and as secondary or absent for classes dominated by non-aromatic polymers such as PE and PP, where hydrophobic and van der Waals interactions instead prevail.

## 5. Comparative Benchmark and Sustainability Considerations

Direct comparison of MNP adsorption performance across studies remains inherently challenging due to pronounced heterogeneity in experimental protocols, and capacities reported by different studies should therefore not be interpreted as directly comparable performance benchmarks. Key variables such as particle type, size, surface chemistry, initial concentration, adsorbent dose, contact time, temperature, pH, ionic strength, and water matrix vary widely, and are often only partially reported [3,31,112]. Despite this heterogeneity, Table 2 presents representative studies, selected to illustrate structurally distinct material subtypes within each and, where available in the literature, to span a range of adsorption capacities and both synthetic and real-water testing conditions; the selection does not imply uniform completeness of parameter reporting across studies, nor systematic coverage of different target polymers, since PS remains the dominant model compound investigated across most classes. The table thus functions as a guiding framework linking adsorption performance to the underlying interaction mechanisms and operating conditions, and the observations drawn from it should accordingly be read as indicative of recurring patterns rather than as statistically representative of each material class as a whole.

Collectively, four consistent trends emerge. First, the highest adsorption capacities are mainly reported for carbon-based and engineered porous materials tested under optimised laboratory conditions. Examples include 3D reduced graphene oxide aerogels with 617 mg g^−1^ for PS, chitin–lignin hydrogels reaching 1790.8 mg g^−1^, Fe-doped magnetic sponge carbon with 369.07 mg g^−1^ in ten minutes, and magnetic carbon nanotubes (M-CNTs) showing 1100–1650 mg g^−1^ across polymers [26,59,108,126]. However, these values are typically obtained in simplified systems based on monodisperse PS in deionised water, which favour π–π interactions and exclude competition from dissolved species, thus overestimating real-world performance [20,31]. Second, the findings suggest a systematic decrease in performance when moving from synthetic to real water matrices. A representative example is the 3D reduced graphene oxide aerogel, whose capacity drops from 617 to 431 mg g^−1^ in micro-polluted lake water [59]. This reduction may be associated with charge screening by ions and competition from dissolved organic matter, which mainly affect electrostatic interactions and pore accessibility, while π–π stacking remains comparatively stable. Similar trends were observed across all material classes. For instance, banana peel adsorbent achieved only 0.085 mg g^−1^ in real effluent despite 97.5% removal [99], highlighting the need to report both capacity and efficiency. Multi-mechanism systems such as D-DPGG aerogels maintain >90% efficiency across different waters [106], and process-level studies already show stable performance above 90% in real WWTP conditions [21,22], indicating growing practical relevance. Third, adsorption kinetics vary widely and reflect the dominant mechanism. Chemisorption-based systems, especially metal-doped or magnetic materials, reach equilibrium within minutes, while physisorption-based materials could require hours due to potential diffusion limitations [26,121,125]. This difference can directly affect process design, with fast systems suited to continuous treatment and slower ones to batch operation [20]. Fourth, the evaluated studies highlight the limited representation of mixed-polymer systems, which are more environmentally relevant. Most studies focus on single polymers, typically PS, while only a limited number consider mixed systems. This bias also affects mechanistic interpretation, often amplifying the perceived importance of π–π interactions compared to aliphatic polymers. PS is aromatic but largely non-polar, and its adsorption is therefore mainly associated with π–π stacking and hydrophobic interactions. In contrast, PE and PP lack aromatic rings and are expected to interact primarily through hydrophobic and van der Waals forces. Polymers such as PVC, PMMA, and PVAc contain polar functional groups (e.g., chlorine or ester carbonyls), which may also enable hydrogen-bonding or electrostatic contributions. PET combines aromatic and polar functionalities and may therefore involve multiple interaction pathways [127]. As a result, adsorption mechanisms identified for PS may not always be directly transferable to other polymers and should ideally be assessed on a case-by-case basis.

A further layer of complexity, which compounds the experimental heterogeneity discussed above, concerns the analytical techniques employed to quantify MNPs in adsorption studies. The measurement platforms reported across the literature include gravimetric weighing, particle-resolved spectroscopic methods such as µ-FTIR, µ-Raman, and LDIR, and mass-based thermal approaches such as Py–GC/MS, each operating with different detection limits, measurable size ranges, and output metrics. Particle-resolved techniques provide particle counts, size distributions, and polymer identity but become less reliable below 10 µm and do not readily yield mass concentrations, while pyrolysis-based approaches deliver polymer-specific burden data but lose morphological and particle-number information [5,128]. Removal efficiencies expressed as particle number, mass concentration, or gravimetric loss are therefore not directly interchangeable, and comparisons between studies using different instruments should be interpreted with caution. Analytical variability is acknowledged as a key factor constraining cross-study comparability in MNP research [3,30]; this should be taken into account when evaluating the adsorption data presented below. Improving consistency in reporting experimental conditions would further enhance comparability across studies.

### 5.1. Cost, Scalability, and Feedstock

Building on the performance trends discussed above, practical implementation requires moving from adsorption efficiency to considerations of cost, scalability, and material sourcing. Some differences should be encountered across the five adsorbent classes, reflecting not only performance but also production pathways and technological maturity.

Among these, bio-based and biowaste adsorbents offer the most evident cost advantage. Agro-industrial residues such as banana peels, sugarcane bagasse, coconut bagasse, and bark derived biochars, among other biomasses, are already available within existing supply chains, and their use as adsorbents adds value without requiring dedicated production. Slow pyrolysis biochar from forestry sidestreams is typically estimated at 200 to 500 US dollars per tonne [87], compared with 1500 to 4000 US dollars per tonne for commercial activated carbon and significantly higher costs for MOFs and COFs. This cost gap is mirrored on the environmental side, with a meta-analysis of life cycle data reporting average energy demands of about 6 MJ per kilogram for biochar production against roughly 97 MJ per kilogram for activated carbon, and corresponding greenhouse gas emissions of about −0.9 and 6.6 kg of CO_2_-equivalent per kilogram, respectively, reflecting the carbon sequestration credit associated with pyrolysis [129]. In addition, this also reflects a deeper distinction, since scaling biochar is primarily an engineering task, whereas scaling advanced materials often remains limited by complex multi-step synthesis at the reactor level.

Carbon-based adsorbents occupy an intermediate position. GAC is already widely used in drinking water treatment [84], making its extension to MNP removal relatively straightforward. In contrast, high-performance variants such as graphene-based aerogels and functionalised carbon nanotubes remain largely confined to laboratory-scale due to higher production costs and environmental burden. Metal hydroxides and magnetic composites show similar variability. Systems based on Fe_3_O_4_ decorated biochar largely retain the cost profile of the underlying biochar [121], while materials such as LDHs require metal precursors that introduce additional cost and supply considerations.

Porous engineered materials including aerogels, sponges, and hydrogels deliver excellent adsorption performance but still lack clear scale-up strategies beyond batch synthesis. At the high-performance end, MOFs and COFs are constrained by synthesis routes that rely on high-purity precursors, solvothermal conditions, and long reaction times, which are difficult to reconcile with large-scale water treatment. In this context, aluminosilicate materials provide a more accessible alternative, combining structural functionality with industrial scalability, as shown by the one year pilot operation of modified zeolite bentonite systems in real treatment conditions [21].

### 5.2. Regeneration Performance

Extending the discussion from cost and scalability, regeneration and end-of-life behaviour provide an additional layer for evaluating the practical viability of these materials.

The choice of regeneration strategy may also be related to the type of interaction involved. Chemisorption, which relies on covalent or coordinative bonds, tends to require comparatively more demanding treatments, such as acid washing or thermal desorption, while hydrophobic and π–π interactions, being generally weaker, can often be reversed through milder solvent washing [130]. Electrostatic attraction and hydrogen bonding may offer some advantage in this respect, as their sensitivity to solution pH and ionic strength can in some cases allow regeneration to be approached by adjusting solution chemistry, although the extent of desorption achieved this way appears to vary depending on the specific adsorbent and pollutant pair [130]. By contrast, pore filling does not involve the disruption of specific chemical bonds during desorption. As a result, regeneration performance may depend more strongly on the mechanical and structural stability of the adsorbent framework, which has been reported as a potential limitation for some hydrogel-based materials during repeated adsorption–desorption cycles [131].

Carbon-based and magnetic composite adsorbents show the most consistent regeneration performance. Capacity retention between about 60% and over 90% after several cycles has been reported for Fe_3_O_4_ modified biochars, Fe doped magnetic sponge carbon, and magnetic CNT composites [26,121,126], typically using solvent washing or mild thermal treatment. Their behaviour reflects the combined role of chemisorption and magnetic recoverability, which allows rapid separation, reuse of solvents, and, when needed, thermal treatment of the captured polymer at high temperature as part of a capture and degradation strategy [132]. MOF-based materials also show promising multi-cycle performance, with efficiencies remaining above 89% after repeated use and stable operation under realistic conditions [114,115]. However, their long-term environmental compatibility is still uncertain, particularly due to the limited assessment of metal leaching during regeneration [20]. In contrast, bio-based and biowaste adsorbents follow a different approach, with regeneration less explored and most studies effectively assuming single use followed by thermal disposal or energy recovery, a strategy that aligns with their low cost but ultimately limits the overall removal efficiency per unit mass. Porous engineered systems occupy an intermediate position, with some aerogels such as D-DPGG maintaining relatively stable performance over repeated cycles, while hydrogels and sponges can generally be reused only a limited number of times with moderate losses in capacity [106], and their longer term application remains constrained by mechanical stability, which is often not systematically assessed. End-of-life management remains one of the least explored aspects, particularly for MOF and COF systems. The lack of systematic data on material stability and potential release of metal species during regeneration represents a significant knowledge gap that must be addressed before large-scale application becomes feasible.

### 5.3. Sustainability and End-of-Life Considerations

From a broader sustainability perspective, biochar- and biowaste-based adsorbents emerge as the most favourable option. This advantage reflects the use of renewable and locally available feedstocks, relatively low energy requirements during synthesis compared with thermochemical or solvothermal routes, and compatibility with end-of-life strategies that close the carbon loop, such as energy recovery or soil amendment, provided that the residual MNP content is properly assessed before disposal [8,87].

One of the most promising near-term opportunities lies in integrating MNP targeted biochar production into existing pyrolysis infrastructure, where the same processes already used for biomass valorisation can be adapted to produce adsorbents with limited additional investment, effectively shifting their role from specialised materials to co-products of established biorefineries.

At the opposite end, graphene-based aerogels and MOFs or COFs remain associated with the highest energy and solvent demand, with life cycle assessments of representative synthesis routes attributing more than 85% of the overall environmental impact to solvent use alone [133], although recent advances suggest that their environmental footprint could be reduced through greener synthesis strategies, including solvent free mechanochemistry [134], biomass-derived precursors [135], and electrochemical routes [136]. Metal hydroxides and magnetic composites show a more balanced profile, combining good scalability with moderate constraints related to the energy cost of metal precursors and, in some cases, the demands of magnetic separation, while their sustainability improves significantly when waste-derived supports such as biochar, fly ash, or mining residues are used, linking adsorption performance to circular resource streams and reducing the material cost per unit of MNP removed.

Across all material classes, the most critical life cycle uncertainty concerns the fate of the captured polymers. If MNPs are not degraded or securely contained, disposal of the spent adsorbent risks simply transferring contamination from water to soil or landfill. In this context, integrated capture and degradation approaches offer a particularly promising direction. Examples include thermal treatment of modified biochars, Fenton-like oxidation in hybrid composites, and photocatalytic degradation in functional membranes, all of which aim to eliminate the polymer rather than redistribute it [116,117,132]. However, their broader application is still limited by uncertainties related to catalyst stability over repeated cycles and to the incomplete characterization of degradation by products, both of which remain key priorities for future research [20].

Taken together, cost, regeneration, and sustainability considerations highlight the trade-offs guiding adsorbent selection. As summarised in Table 3, different adsorbent classes offer distinct advantages and limitations, with no material consistently outperforming others across all criteria. Consequently, adsorbent selection should consider the specific application and operating context rather than adsorption capacity alone.

## 6. Conclusions

This review has examined the adsorption of MNPs in water from a mechanism-oriented perspective, linking molecular interactions to the structural features of the main classes of adsorbents.

Overall, adsorption emerges as a system-dependent process governed by the interplay between polymer properties and surface chemistry rather than by the material alone. Interactions such as π–π stacking and hydrophobic affinity dominate for non-functionalised polymers on carbon-rich surfaces, while electrostatic forces and hydrogen bonding become more relevant for oxidised particles. Structural factors such as pore architecture further modulate uptake when matched to particle size, whereas chemisorption provides a stronger and faster pathway in metal-containing systems.

Across material classes, differences are more closely related to scalability, tunability, and sustainability than to intrinsic adsorption capacity. Biochar and bio-based materials offer a favourable balance between performance and practical implementation, particularly due to their compatibility with existing production systems. More advanced materials such as MOFs, COFs, and graphene-based structures enable greater mechanistic control but remain constrained by synthesis complexity and cost.

Importantly, the comparison section highlights that adsorption capacities measured under simplified laboratory conditions could overestimate performance in real systems. Removal efficiency under realistic, multi-component conditions therefore represents a more meaningful metric for comparison across studies.

Despite recent progress, key challenges remain. The behaviour of NPs under environmentally relevant conditions is still poorly resolved, and most studies continue to focus on PS as a model system due to its ease of detection and its strong affinity for common adsorbents, which limits representativeness. Experimental protocols are not yet standardised, and regeneration as well as end-of-life pathways require further validation, particularly for materials involving metal components. Moreover, a transition toward studies in real water matrices and at larger scale, including column and pilot systems, is still needed to better capture competitive effects, transport limitations, and long-term performance.

Future advances will likely depend on closer integration between modelling and experimental approaches, including multiscale simulations linking molecular-level interactions to macroscopic performance, and operando spectroscopic techniques capable of tracking adsorption mechanisms under realistic, dynamic conditions. Machine learning and AI-assisted approaches are also expected to accelerate adsorbent design and the prediction of adsorption performance across polymer types and water matrices, an application already demonstrated using random forest, support vector machine, and neural network models to predict microplastic–water partitioning behaviour [137]. On the application side, progress will depend on the development of multifunctional systems that combine capture with degradation, hybrid adsorption membrane configurations, and pilot-scale continuous reactors that allow adsorption processes to be incorporated into existing water treatment infrastructures. Continued validation under realistic conditions will be essential to translate laboratory-scale advances into reliable field applications.

## Figures and Tables

**Table 1:** Comparison of the organising principle and mechanistic scope of representative reviews on MNP adsorption.

Organising Principle	Mechanistic Treatment	Cross-Mechanism Comparison (≥3 Classes)	Relation to Present Review	Ref.
Bibliometric and technique-based	Mechanisms (electrostatic, hydrogen bonding, π–π, hydrophobic, chemisorption) summarised generically in an overview section	No	Mechanisms are surveyed narratively, not compared consistently across adsorbent classes	[3]
Material class-based (carbon materials, sponge/aerogel/fiber, metal (hydr)oxides, MOFs)	Mechanisms discussed within each material subsection	No	Same mechanisms recur across sections without cross class comparison	[4]
Material class-based, within bio-based adsorbents (biochar, biomass-derived activated carbon, sponges/aerogels, graphene-based materials)	Mechanisms of MP–bioadsorbent interaction discussed narratively at the end	No	Confined to biomass-derived adsorbents; mechanisms are a closing narrative, not the organising framework	[8]
Material class-based, within porous materials (sponge/aerogel/hydrogel, MOFs, metal-based materials, carbon-based materials)	Proposed mechanism tabulated per individual adsorbent, not distilled into class level trends	No	Broader material coverage but limited to porous materials; mechanisms recorded per adsorbent rather than synthesised across classes	[20]
WWTP treatment stage-based	Physical separation and sedimentation, not molecular interactions	No	Does not address molecular-scale adsorption mechanisms	[17]
Broad narrative review connecting MP pollution, toxicology, and waste management with adsorption technology	Adsorbent design and performance discussed narratively; no dedicated mechanism-based framework	No	Wider disciplinary scope but a shallower, non-systematic treatment of mechanisms	[14]
Mechanisms and adsorbent classes (carbon materials, MOFs, inorganic materials) treated as separate, independent surveys	Six mechanisms illustrated generically (electrostatic, hydrophobic, π–π, pore filling, complexation, hydrogen bonding); not mapped back to specific material classes	No	Nearly identical mechanism set to the present review, but mechanisms are never attributed to specific adsorbent classes; distinctive focus on column and continuous flow adsorption and predictive simulation models	[27]
Mechanism-first, but confined to one material class	Hydrophobic, hydrogen bonding, van der Waals, electrostatic, π–π, pore filling, intraparticle diffusion, applied generically before per material behavioural analysis	Partial (carbon materials only)	Confirms that a mechanism-first organisation is valuable, but applies it only to carbon-based adsorbents, without cross class extension	[28]

**Table 2:** Representative adsorption performance data for the five adsorbent classes (N.R. = data not reported; HDPE = high-density polyethylene; some values are isotherm-derived maxima).

Material Class	Adsorbent	Target Polymer	MNP Size	Adsorption Capacity (mg g^−1^)	C_0_ (mg L^−1^)	Adsorbent Dose (g L^−1^)	Solution Volume (mL)	Removal Efficiency (%)	t_eq (min)	Matrix	Dominant Mechanisms	Analytical Method	Ref.
Carbon-based	Coconut shell-based GAC	PS	90 nm	6.33	40	5	30	~90	240	Natural surface water (Geneva lake)	Electrostatic interactions, chemisorption, pore filling	Turbidimetry (mass basis)	[84]
Carbon-based	3D reduced graphene oxide	PS	5 µm	617.28, 430.78	600	0.6	2	>53 (real matrix)	120	Distilled water, micro-polluted lake water	π–π stacking, pore filling, electrostatic interactions, chemisorption	UV-Vis (mass basis)	[59]
Carbon-based	Oxidized corncob biochar	PS	50 nm	23.98	400	20	10	>90%	1440	Synthetic	Pore filling, hydrophobic interactions, H-bonding, electrostatic repulsion (low pH)	UV-Vis (mass basis)	[90]
Bio-based	Banana peel biowaste	PE, PP, PET, PS (mixed)	1.5–135 µm	0.085, 0.078	N.R.	0.3	1000	99, 97.5	25	Synthetic, real WWTP effluent	Electrostatic interactions, H-bonding, chemisorption, hydrophobic interactions/van der Waals (PE, PP), π–π stacking	Optical microscopy and FTIR (mass and particle number basis)	[99]
Bio-based	Tween-80-modified sugarcane bagasse	PVC, PET, PS, PP, HDPE (mixed)	<600 µm	N.R.	1200	5	100	80	2880	Synthetic	Hydrophobic interactions, H-bonding	FTIR (mass basis)	[100]
Bio-based	Surface-modified cellulose fibers	PVC, PMMA, Polyvinyl acetate (PVAc)	~100 nm	N.R.	2000	2.2	6	>98	30	Synthetic	Electrostatic interactions, H-bonding	UV-Vis (mass basis)	[61]
Porous Engineered Materials	Chitin–lignin hydrogel	PS	<1 µm	1790.8	1000	N.R.	N.R.	N.R.	240	Synthetic	Electrostatic interactions, π–π stacking	UV-Vis (mass basis)	[108]
Porous Engineered Materials	Graphene oxide/nanocellulose aerogels (D-DPGG)	PS	1 µm	241.56	100	N.R.	N.R.	96.62	20	Synthetic; river, lake, reservoir, marine water	Electrostatic interactions, π–π stacking, pore filling, H-bonding	Fluorescence spectrophometer (mass-based)	[106]
Porous Engineered Materials	Starch-gelatin sponge	PMMA, PS	100 nm, 5 µm	20.43, 21.79	25	1.25	40	N.R.	1440	Synthetic; tap water, seawater, soil supernatant, take-out dish soup	Pore filling, H-bonding, hydrophobic interactions	Fluorescence spectropho-tometer (mass-based)	[125]
MOFs/COFs	Fe_3_O_4_@MP-COF/PVDF	PE, PP, PS	75–150 µm	280, 380, 520	50	0.1	50	N.R.	~40–60	Synthetic	π–π stacking, electrostatic interactions	Gravimetric analysis (mass basis)	[117]
MOFs/COFs	ZIF-67	PS	1 µm	11.6	5	0.4	30	92.1	20	Synthetic	Electrostatic interactions, π–π stacking, H-bonding	Fluorescence spectrometry (mass basis)	[63]
MOFs/COFs	Chromium-based metal–organic framework (Cr-MOF/MIL-101)	PS	50–70 nm	800	5–100	0.1	40	96	1440	Synthetic	Electrostatic interactions	UV-Vis (mass basis)	[112]
Metal (hydr)oxides and magnetic composites	M-MgAl-LDO	PS	80 nm	203.12	200	1	10	97	360	Synthetic	Chemisorption/surface complexation, pore filling, π–π interactions, electrostatic interactions, hydrogen bonding	Fluorescence spectrometry (mass basis)	[122]
Metal (hydr)oxides and magnetic composites	Fe_3_O_4_-modified biochar	PS	1 µm	290.20	10	0.5	10	N.R.	30	Synthetic	Chemisorption/inner-sphere complexation, electrostatic interactions	Fluorescence spectrometry (mass basis)	[121]
Metal (hydr)oxides and magnetic composites	Fe-doped magnetic sponge carbon (FeMSC)	PS	>1 µm	369.07	200	0.5	20	>90	10	Synthetic	Chemisorption, π–π stacking, magnetic separation	Fluorescence spectrometry (mass basis)	[26]

Reported capacities for Magid et al. [78] and Modak et al. [100] derive from Langmuir isotherm fits obtained over a wider concentration range than the C_0_ value used for kinetics or removal efficiency in the same study.

**Table 3:** Comparative summary of the five adsorbent classes for MNP removal, highlighting relative production cost and scalability, regeneration performance over reuse cycles, sustainability profile, and the main limitation currently constraining practical application of each class.

Material Class	Cost & Scalability	Regeneration	Sustainability	Main Limitation
Carbon-based	Moderate–high (GAC ≈ commercial AC, 1500–4000 USD/t); advanced forms lab-scale only	High, 60–90% over cycles	High footprint for graphene aerogels; conventional forms not quantified	Performance–cost trade-off
Bio-based and biowaste	Lowest cost (200–500 USD/t), highly scalable	Limited, mostly single-use	Most favourable, renewable, low-energy, circular	Low reuse limits efficiency per mass
Porous engineered materials	High performance, poor scale-up beyond batch	Intermediate, moderate capacity loss	Mixed: biodegradable, bio-based hydrogels favourable; aerogel/sponge drying energy- and solvent-intensive	Scale-up and mechanical stability
MOFs/COFs	High cost, complex synthesis; aluminosilicates cheaper, proven	High, >89% retained	Highest footprint; greener routes emerging	Metal leaching, long-term compatibility unclear
Metal (hydr)oxides and magnetic composites	Variable, tied to support cost	High, 60–90%, magnetic recovery	Balanced; improves with waste-derived supports	Oxidation, metal leaching on cycling

## Data Availability

No new data were created or analyzed in this study. Data sharing is not applicable to this article.
